# Developing a Customizable Texting Intervention for Diabetes Self-Management: Participatory Design Approach

**DOI:** 10.2196/83144

**Published:** 2026-01-13

**Authors:** Stephanie A Robinson, Popy Shell, Linda Am, Courtney L Bilodeau, Howard S Gordon, Constance R Uphold, Varsha G Vimalananda, Sarah L Cutrona, Timothy P Hogan, Bridget Smith, Stephanie L Shimada

**Affiliations:** 1 Center for Health Optimization and Implementation Research VA Bedford Healthcare System Bedford, MA United States; 2 The Pulmonary Center Boston University School of Medicine Boston, MA United States; 3 Malcolm Randall Department of Veterans Affairs Medical Center Gainseville, FL United States; 4 Jesse Brown Department of Veterans Affairs Medical Center Chicago, IL United States; 5 Department of Academic Internal Medicine University of Illinois College of Medicine Chicago, IL United States; 6 Department of Physiology and Aging College of Medicine University of Florida Gainesville, FL United States; 7 Section of Endocrinology, Diabetes, Nutrition and Weight Management Department of Medicine Boston University Chobanian and Avedisian School of Medicine Boston, MA United States; 8 Department of Population and Quantitative Health Sciences University of Massachusetts Medical School Worcester, MA United States; 9 Department of Population and Quantitative Health Sciences UT Southwestern Medical Center Worcester, MA United States; 10 VA Hines Healthcare System US Department of Veterans Affairs Hines, IL United States; 11 Department of Health Law, Policy, and Management Boston University School of Public Health Boston, MA United States

**Keywords:** text messaging, type 2 diabetes, veterans, mobile phone, self-management

## Abstract

**Background:**

Uncontrolled diabetes contributes to serious comorbidities and mortality. Effective self-management can improve outcomes, though barriers such as limited education and support often prevent patients from engaging in such behaviors. Automated texting systems show promise to deliver diabetes self-management education as they are accessible and scalable. Furthermore, customizing these systems may further enhance patient engagement compared to standard, one-size-fits-all approaches. However, such customization is more resource-intensive, and it remains unclear whether the added effort meaningfully enhances diabetes self-management and outcomes.

**Objective:**

This study aimed to describe the development of 2 versions of an automated texting system intervention for diabetes self-management: (1) a standard, education-only intervention (Diabetes Self-Management Support; DSMS) and (2) an interactive, customizable intervention (Diabetes Self-Management Support + Interactive and Customizable Messages; DSMS+).

**Methods:**

Two versions of an automated texting system intervention were developed using a participatory design approach that incorporated input from veterans and expert clinicians. Message content was refined through feedback from a multidisciplinary team, veteran coinvestigators, national surveys, interviews, clinical expert panel reviews, and beta testing. Surveys were mailed to 1000 potential participants, oversampling rural, low-income, minority, and female participants. Respondents rated message relevance and provided preferences for content, timing, and frequency. Interviews provided customization preferences. A clinical expert panel reviewed all messages for safety and appropriateness. Beta testing informed final refinements.

**Results:**

Ninety-two surveys were completed (9.2% response rate). Respondents rated 62% of the messages as personally relevant and 61% confidence-enhancing. Interviews with 23 respondents revealed a preference for 1-2 texts per day, emphasizing topics such as healthy eating and weight management. The clinical expert panel reviewed 536 messages, flagging 81 for revision. Beta testing confirmed feasibility and informed refinements to clarity and timing. The 2 resulting interventions were built in the US Department of Veterans Affairs’ automated texting system, Annie.

**Conclusions:**

Two text messaging interventions, DSMS and DSMS+, were developed to support diabetes self-management among US veterans. DSMS delivers standard educational content, while DSMS+ incorporates interactive features and personalization. The subsequent clinical trial will assess whether customization enhances engagement and improves diabetes outcomes, providing insights into the potential of tailored mobile health interventions for chronic disease management.

## Introduction

### Background

Diabetes is a serious chronic health condition that can exacerbate multiple comorbidities and increase the risk of death [1]. This disease affects nearly 15% of US adults and almost 25% of US veterans [2,3]. Furthermore, diabetes is disproportionately prevalent in minority groups and in those from low socioeconomic status, particularly males from minority groups and females from low socioeconomic status [4]. Effective diabetes self-management behaviors, such as healthy eating, exercise, medication adherence, and glucose control, can significantly improve health outcomes and quality of life [5,6]. There is evidence that programs such as self-management interventions and diabetes education may improve outcomes. However, there is a need to increase the reach and sustainability of these programs [7].

Automated texting systems (ATSs) have become more popular as a delivery method for diabetes self-management education for several reasons [7]. For one, they have demonstrated effectiveness in promoting a wide range of healthy behaviors [8-10] across a range of diseases, such as HIV, chronic obstructive pulmonary disorder, cardiovascular disease, and asthma [11]. ATS can reinforce healthy behaviors by providing reminders and offering disease education, resulting in improved outcomes [12-14]. One meta-analysis reported significantly better glycemic control post-ATS intervention [15]. Additionally, given the ubiquity and relatively low cost of cell phones compared to more complicated digital health interventions, ATS likely do not require as much digital literacy and they are relatively accessible and scalable [7].

The US Department of Veterans Affairs (VA) has a proprietary ATS, called “Annie,” named after Veteran Annie Fox who was the first nurse to be awarded a Purple Heart. VA’s Annie is a disease-agnostic SMS texting system that can send 1-way messages and receive and respond to patient messages if the messages follow specific syntax. Responses to 2-way messages use rule-based branching logic [16,17]. Previous research has shown that Annie improved patient self-management for the hepatitis C virus [16]. Annie has also been used to deliver educational content related to COVID-19 (precautions and monitoring symptoms). Veterans reported that receipt of Annie’s messages prevented them from contacting VA by phone, secure message, or visiting, thus saving VA resources [18]. Messages from an Annie intervention for caregiver stress management resulted in caregivers reporting that they felt more cared for and more confident [19]. Annie has also been leveraged to be customized to the needs of specific subgroups, such as hypertension management in African American veterans [20]. While there are currently thousands of veterans actively using Annie, this is just a small percentage of US veterans and work is needed to understand how to make these systems as engaging as possible so that users will want to adopt and continue using them.

Customizing the text messaging intervention can increase its impact by addressing individual needs and preferences. Tailoring content and timing to each patient’s unique circumstances, such as their current knowledge level, specific challenges and schedules, and personal goals, makes interventions more relevant and engaging. Personalization fosters greater autonomy and empowerment, increasing the likelihood of active engagement and adherence to self-management strategies, ultimately improving health outcomes [21]. Past implementation evaluations of Annie found that the ability to tailor ATS content and timing encouraged patient use and enhanced patient autonomy [17]. While a customized intervention can be resource-intensive to develop, the effort may be worthwhile for patients needing more individualized support. In contrast, a standard intervention may be less resource-intensive and easier to implement at scale, but it may not fully address the unique needs of each patient, potentially limiting its effectiveness for those with diverse or complex challenges. Indeed, a recent review suggests that ATS programs with daily messages and additional features to support customization were more effective in diabetes outcomes than interventions that had infrequent, fully automated messages [7]. A recent systematic review and meta-analysis of tailored text messaging interventions for type 2 diabetes highlighted significant limitations in prior studies [12]. Specifically, most previous work has tested these personalized interventions in high-income settings and among patients with relatively well-controlled diabetes. These populations are less likely to represent those who could benefit most from additional support.

### Current Study

This study details the development of 2 versions of a text messaging intervention for diabetes self-management that are uniquely focused on developing content designed to be tailored to US veterans who face greater challenges in achieving glycemic control, particularly in more diverse and underserved settings. The first version is a standard, education-only intervention. The second is an interactive, customizable texting self-management intervention developed through a participatory design process that incorporates veteran preferences, clinician input, and evidence on effective texting self-management modules. This study details the development of these interventions as part of a larger study aiming to compare the effectiveness of these 2 interventions on diabetes control.

## Methods

### Platform

**Figure 1:**
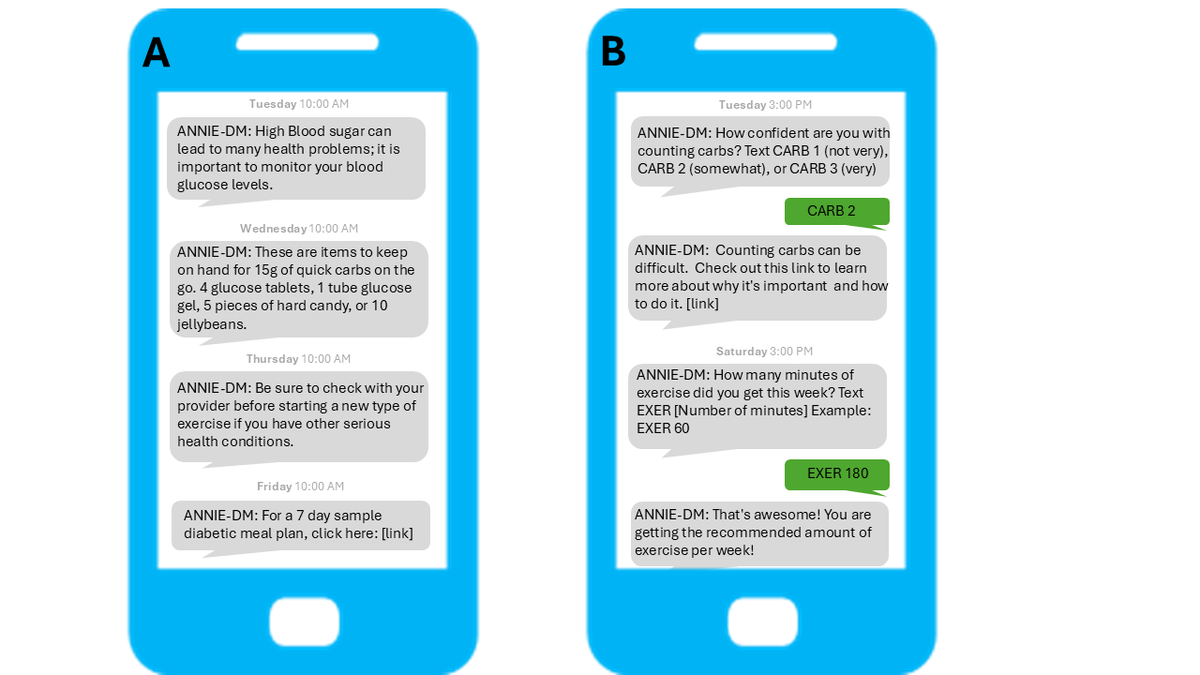
Sample messages from the 2 diabetes self-management automated texting system interventions, developed for the Annie platform within the Veterans Health Administration. Panel A shows sample 1-way educational messages. Panel B shows sample 2-way messages between the automated texting system and the patient.

The Annie texting system is a mobile messaging service designed to support veterans by sending automated, health-related text messages [22]. These messages provide reminders, education, and encouragement for managing various health conditions, including diabetes, to promote better self-care and adherence to treatment plans. Annie can send 1-way messages, which do not solicit a response, and 2-way messages, which ask the user to respond (Figure 1). The timing and content of messages are flexible. Each message is capped at 160 characters and must include “Annie” as an identifier. Short URLs can also be incorporated. For 2-way messaging, responses must be closed-ended and preprogrammed into the system using keywords.

**Figure 2:**
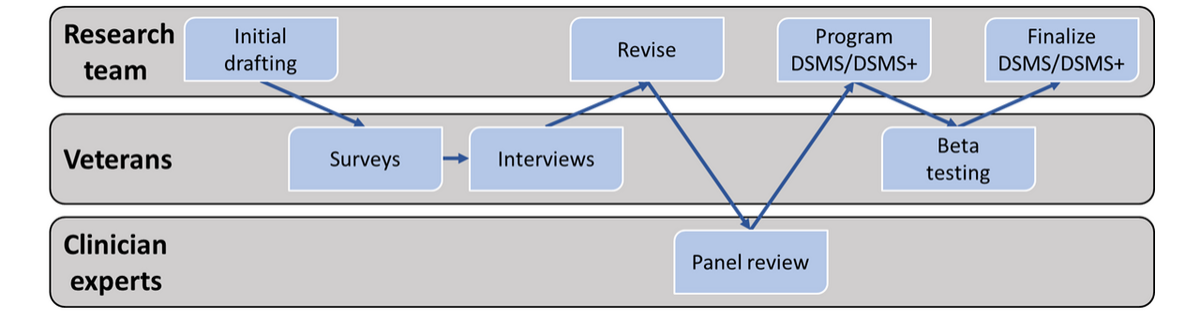
Diabetes self-management automated texting system intervention development stages and stakeholder involvement. The research team included health services researchers, veteran coinvestigators, and clinicians. Veterans included Veterans Affairs patients living with diabetes. Clinician experts included Veterans Affairs and non-Veterans Affairs clinician experts, of which 4 were on the research team. DSMS: Diabetes Self-Management Support-Only texting intervention; DSMS+: customizable, patient-centered version of DSMS.

Below we describe our participatory design approach to developing 2 versions of a text messaging intervention in Annie within the US Veterans Health Administration: a standard Diabetes Self-Management Support-Only Intervention (DSMS) and a customizable, patient-centered intervention (DSMS+); refer to Figure 2. Intervention development drew on the Behavior Change Technique Taxonomy (version 1.0), which integrates overlapping behavioral theories, including Social Cognitive Theory and Self-Determination Theory. These theories discuss the association between patient autonomy, intrinsic motivation, and self-efficacy to support patient engagement and health outcomes [13,23,24]. Text messages were initially drafted by a multidisciplinary research team and then iteratively refined through feedback from veterans and a clinical expert panel review. The final DSMS and DSMS+ interventions were programmed into the Annie texting system by the study team, reviewed for programming errors by the VA Office of Connected Care staff, and finalized. Veterans’ feedback was gathered throughout the development process from veteran coinvestigators, surveys, qualitative interviews, and beta testing.

### Initial Drafting

A multidisciplinary team worked together to draft text messages for use in the 2 versions of our interventions. This team included health services researchers, clinicians, and veterans living with diabetes (“veteran coinvestigators”). Our team met weekly to develop and review the text messages, identify the most relevant and appropriate ones, and iteratively edit them for character count and content.

Text message development for both DSMS and DSMS+ began with modules outlined in standard diabetes educational materials. Initial message and content creation used established diabetes guidelines from updated VA and Department of Defense Self-care Skills for Patients with Diabetes [25], the Association of Diabetes Care and Education [26], and existing Annie protocols (Weight Management Protocols, Blood Glucose Monitoring, and the Blood Pressure 2-way messaging protocols [20]). Most of this content centered on disease education and encouragement to be an active member of the health care team. Our team collated and linked resources to educational materials from the National Institutes of Health, Centers for Disease Control and Prevention, Mayo Clinic, Veterans Health Administration, Food and Drug Administration, American Diabetes Association, US Department of Agriculture, Association of Diabetes Care and Education Specialists, and Department of Health and Human Services. These links were added to relevant text messages. In the end, we developed 160 messages designed to be delivered over the 6 months of the study period. All messages were written aiming for a 5th-grade reading level as recommended by the Joint Commission [27,28].

As part of the research team, veteran coinvestigators were invited to help design the Annie interventions. Three veteran coinvestigators were recruited through VA clinics from 2 VA facilities, though one later dropped out due to timing difficulties. To be eligible, veterans needed a diagnosis of type 2 diabetes and current care through the VA health system. As working members of the research team, veteran coinvestigators were offered hourly compensation for their time. They provided initial feedback on whether the texts were motivating, useful, or needed removal or rewording. Their insights on veteran culture, living with diabetes, and the phrasing and delivery of the messages were invaluable. They also wrote some messages for the “Vet Tips” module to be delivered to other veterans living with diabetes.

### Survey

National feedback from veterans was obtained via surveys. Veterans living with diabetes who owned cell phones were identified using the VA Corporate Data Warehouse, which provides centralized electronic health record data across all VA facilities nationwide. To ensure adequate representation from populations that are often underrepresented in veterans research and disproportionately affected by diabetes, we purposefully oversampled women, rural, minority and low-income veterans. This oversampling captures perspectives from those facing unique barriers to care and self-management, thus improving generalizability. We initially selected 400 participants, oversampling women, Black, rural, low-income veterans, and patients with a mental health diagnosis within the last 2 years. Low income was identified as the bottom quartile of the median household income, identified by ZIP code areas, as reported in the American Community Survey conducted in 2019 [22]. Due to lower responses from Black, low-income, and those with mental health diagnoses, we oversampled these groups by adding 600 more potential participants. Our final list of 1000 was 25% female, 40% Black, 25% rural, 40% low income, and 41% had a mental health diagnosis within the last 2 years.

Eight versions of the survey were developed, each containing different randomly selected text messages. Surveys were randomly sent to the final list of 1000 potential participants and administered between June and November 2021. They reported their demographics and previous engagement in text messaging. They also rated their comfort across 13 technology-related behaviors (eg, checking email and using the internet) on a scale from 0 (not at all comfortable or confident) to 5 (very comfortable or confident), with an option to indicate “not applicable.” Scores on comfort levels (scored from 0 to 5) were averaged across behaviors.

Participants were asked about their preferences for message timing, frequency, and topics (eg, reminders for checking blood sugar and blood pressure, physical activity tips, diabetes coping, healthy eating, etc). They also rated their confidence across 10 diabetes self-management behaviors on a scale from 1 (totally confident) to 5 (not at all confident), with an option to indicate “not applicable,” which was not factored into the mean composite score.

Survey participants were invited to create their own diabetes-related text messages and to react to a set of 25 different messages, indicating “yes” or “no” if they felt the message was personally relevant or made them feel more confident in managing their diabetes. They could also provide edits to messages they did not like. Survey responses were used to refine our messages, design sample calendars, identify questions for follow-up interviews, and select a diverse group of participants for interviews. Participants also had the option to write their own messages for other veterans, offering encouragement, tips that worked for them, helpful information for managing diabetes, or recommended online resources. These messages were combined with ones written by veteran coinvestigators for the Vet Tips module.

### Interviews

In-depth interviews (n=23) were conducted to elicit qualitative feedback on text message content, frequency, and timing. Survey respondents who indicated they were willing to participate in an interview were contacted by phone up to 2 times to schedule the interview. The interviews were recorded using Microsoft Teams. There was one main interviewer and one notetaker who filled out a data collection form based on the participants’ responses.

**Figure 3:**
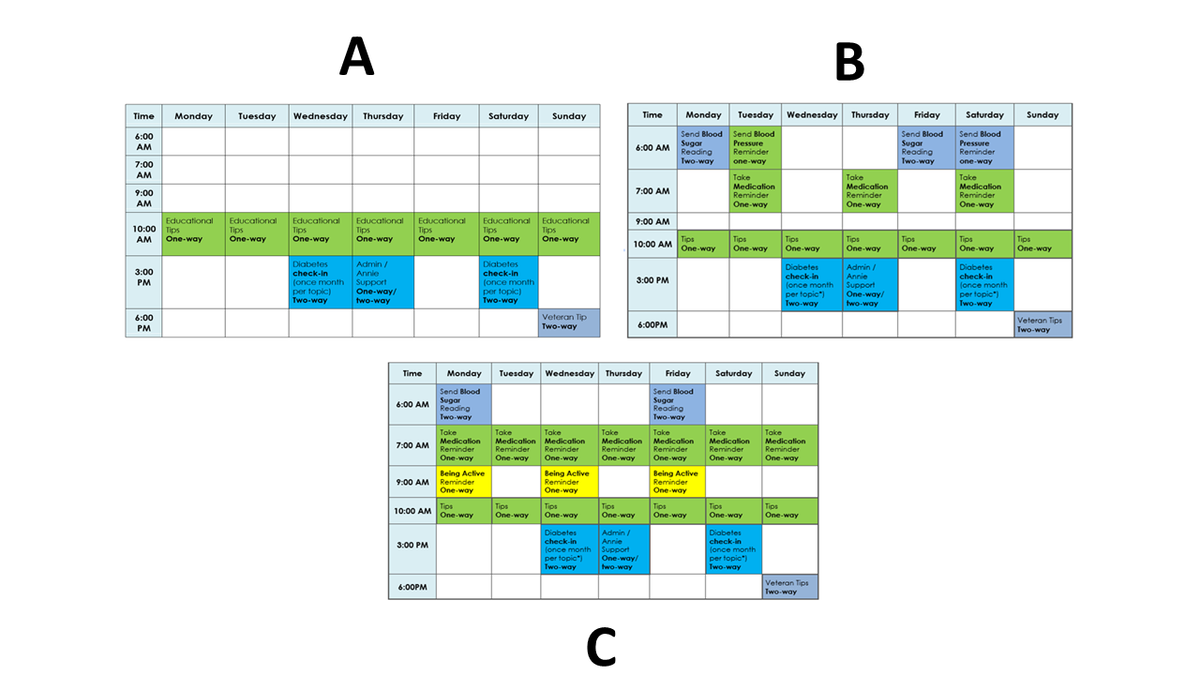
Sample weekly schedules for Diabetes Self-Management Support + Interactive and Customizable Messages, a diabetes self-management automated texting system intervention, developed for the Annie platform within the Veterans Health Administration. Participants asked which panel they preferred, with each showing a range of frequencies and timing of 1-way and 2-way messages.

The interviews followed the introduction of the proposed text messaging program. Types of text messages (eg, 1-way or 2-way) and sample calendars were shown to participants on the types of text messaging schedules that were available (Figure 3). Participants were asked to rank their interest in example text messages on a scale from 1 (not interested at all) to 5 (very interested) and whether the messages addressed specific struggles in managing their diabetes. They also provided feedback on text messages written by survey participants and on hypothetical messaging schedules.

### Clinical Expert Panel Review

In June 2022, a group of 4 VA and 4 non-VA clinician experts, including 4 members of the research team (SC, VV, HG, and CU), gathered virtually for a clinical expert panel. The panel included physicians and nurse practitioners with expertise in endocrinology, primary care, nursing, and obstetrics and gynecology. This panel was tasked with reviewing, rating, and discussing the safety and appropriateness of the text messages to be used in the 6-month randomized control trial. One VA and one non-VA clinician were paired together to review, rate, and discuss 2-3 diabetes-related message topics each. These clinical experts were also asked to independently flag any texts they thought would be problematic from a clinical standpoint. They were all provided with a structured template for feedback and asked to meet with their paired reviewer virtually at another time to discuss any conflicting messages. The experts delivered their final recommendations about which messages to use, drop, or discuss further to the larger team.

### Beta Testing

To identify potential beta testers, we randomly selected 50 patients who met the same eligibility criteria as the planned comparative effectiveness trial. Eligibility criteria included being a veteran and active Veterans Health Administration patient with type 2 diabetes (at least 4 outpatient encounters in the previous year and a future appointment scheduled), access to a smartphone or cellphone, not hospitalized or institutionalized, did not participate in the text message development survey or interview, had at least 2 hemoglobin A_1c_ (HbA_1c_) lab values in the 12 months prior to recruitment, and had inadequate glycemic control for at least half of the 6 months prior to recruitment. These eligible participants were invited by mail and then contacted by the study coordinators up to 3 times by phone to further screen for eligibility and interest and schedule a baseline visit. This screening excluded anyone without access to a cellphone or smartphone, those not willing to text, those with visual impairment that would prevent them from reading or replying to text messages, and those with cognitive impairment as identified by the Short Blessed Test [29]. Phone screening took 6-15 minutes to complete.

Of the 50 potential participants, 33 declined, 8 were never reached (7 were left a voicemail and 1 was an invalid phone number), and 9 were screened (all eligible). All 9 eligible were scheduled for a baseline in November-December 2022. Seven participants ultimately enrolled. These participants were assigned to beta test the DSMS+ Annie message subscription, testing the experience of selecting which support messages they wished to receive for a customized experience. Beta testers also completed all trial procedures, such as baseline and follow-up survey completion, to ensure readiness for the larger trial. Veterans were invited to participate in a follow-up interview and 6 chose to do so. Messages were further refined based on their feedback.

### Ethical Considerations

This study was approved by the VA Central Institutional Review Board (IRB; Project Number 19-30). The study received an IRB waiver of documentation of informed consent. Survey participants were provided with an information sheet with the mailed surveys. Interview and beta testing participants provided verbal informed consent before interviews and beta testing enrollment. All participants were adults and understood participation was voluntary, meaning they could withdraw at any time and skip any question without penalty. Mailed paper survey data were entered into a VA-approved secure web platform for managing survey data; beta testers were also able to complete surveys electronically so that data were directly captured. Interviews were conducted in Microsoft Teams. Recordings and transcriptions were immediately exported and stored on a server that only IRB-approved research team members could access. All electronic data were stored behind the VA firewall and accessible only to IRB-approved research team members. A unique study ID was assigned to each veteran participant with an identification key kept in a separate file accessible only to team members with a need to access. Participants were compensated with gift cards of US $25 for the survey, US $30 for the interview, and US $50 for beta testing.

## Results

### Survey

Out of the 1000 surveys mailed, 92 were returned fully completed (9.2% completion rate). We received surveys from 8 to 17 participants per version, totaling 92. Mean age was 64.5 (SD 7.2) years, ranging from 45 to 76 years. Of the 92 respondents, 63.0% (n=58) were male, 42.4% (n=39) African American or Black, and 28.3% (n=26) White. Additionally, 82.6% (n=76) did not consider themselves to be Hispanic or Latino, 67.4% (n=62) lived in an urban area, and 40.2% (n=37) had a bachelor’s or associate’s degree. More demographic details are listed in Table 1. Regarding familiarity with technology and texting, the majority (84.8%, n=78) reported they had sent or received text messages. Participants’ mean comfort in technology was 3.8 (SD 1.6), where a number closer to 0 indicates less comfort and a number closer to 5 indicates greater comfort. Participants’ mean confidence in diabetes self-management was 2.08 (SD 0.81), where a number closer to 1 indicates less confidence and a number closer to 5 indicates greater confidence.

**Table 1:** Demographic characteristics (N=92) of survey participants for the formative development of 2 diabetes self-management automated texting system interventions designed for the Annie platform within the Veterans Health Administration.

Demographic characteristics			Values
Age (years), mean (SD)			64.53 (7.23)
**Sex,^a^n (%)**			
	Female	34 (37.0)	
	Male	58 (63.0)	
**Race,^a^ n (%)**			
	African American or Black	39 (42.4)	
	American Indian or Alaska Native	11 (12.0)	
	Asian	3 (3.3)	
	Native Hawaiian or Other Pacific Islander	12 (13.0)	
	White	26 (28.3)	
	Unknown	1 (1.1)	
**Ethnicity, n (%)**			
	Hispanic or Latino	7 (7.6)	
	Not Hispanic or Latino	76 (82.6)	
	Missing	9 (9.8)	
**Living area^a^, n (%)**			
	Rural	30 (32.4)	
	Urban	62 (67.4)	
**Education, n (%)**			
	High school graduate or GED^b^ or less	18 (19.6)	
	Some college or vocational school (1-4 years)	24 (26.1)	
	Bachelor’s or associate’s degree	37 (40.2)	
	Master’s or Professional school degree	9 (9.8)	
	Missing	4 (4.3)	
**Employment, n (%)**			
	Employed full-time	14 (15.2)	
	Employed part-time	8 (8.7)	
	Not employed, looking for work	1 (1.1)	
	Not employed, not looking for work	0 (0.0)	
	Retired	38 (41.3)	
	Disabled, not able to work	36 (39.1)	
	Missing	3 (3.3)	
**Relationship status, n (%)**			
	Married, civil union, engaged, or in a relationship	49 (53.3)	
	Divorced, separated, or widowed	22 (23.9)	
	Single, never married or in a civil union	18 (19.6)	
	Missing	3 (3.3)	
**Low income^a^ (bottom quartile of median household income, identified by ZIP code), n (%)**			
	No	52 (56.5)	
	Yes	40 (43.5)	
**Perceived difficulty paying for basics like food and heating or cooling, n (%)**			
	Very hard	7 (7.6)	
	Hard	6 (6.5)	
	Somewhat hard	31 (33.7)	
	Not very hard	38 (41.3)	
	Don’t know	4 (4.3)	
	Prefer not to answer	5 (5.4)	
	Missing	1 (1.1)	
**Diabetes care location, n (%)**			
	I get all my diabetes care in the VA^c^	66 (71.7)	
	I get most of my diabetes care in the VA, with some outside care	14 (15.2)	
	I get most of my diabetes care outside the VA, with some care in the VA	6 (6.5)	
	I get all my diabetes care outside the VA	3 (3.3)	
	Missing	3 (3.3)	
**Mental health diagnosis within last 2 years,^a^ n (%)**			
	No	47 (51.1)	
	Yes	45 (48.9)	

^a^Data extracted from the VA Corporate Data warehouse, all other data self-reported via survey.

^b^GED: General Educational Development.

^c^VA: Veterans Affairs.

Across all 8 versions of the survey, containing a unique combination of 25 messages, participants rated 62% of the messages as personally relevant. Additionally, 61% of the messages made them feel more confident that they could manage their diabetes (range across surveys: 46.7%-78.8%). The most popular reminder topics were reminders to measure blood sugar, measure blood pressure, and exercise (Table 2). The most popular tips related to managing blood sugar, coping with diabetes, being active, and eating healthy.

**Table 2:** Frequency of survey participants (N=92) who endorsed specific topics for inclusion during the formative development of 2 diabetes self-management automated texting system interventions developed for the Annie platform within the Veterans Health Administration.

Topic		Participants, %
**Reminders**		
	Reminder to check blood sugar	52.2
	Reminder to check blood pressure	40.2
	Reminders to take medications	30.4
	Physical activity reminder	38.0
**Tips**		
	Tips for managing blood sugar	55.4
	Tips for coping	52.2
	Tips for healthy eating	51.1
	Tips for being active	51.1
	Tips for setting health goals	47.8
	Tips for managing weight	46.7
	Other educational topics	46.7
	Tips for managing blood pressure	40.2
	Tips for managing medications	38.0

For each topic, participants were asked to choose how many days per week and at what times they would prefer to receive each message. Respondents could choose a default time (eg, 9 AM or 3 PM), a custom time, or indicate no preference. The majority preferring blood sugar and medication reminders requested customization (56% for blood sugar reminders, 60% for diabetes medication reminders, and 64% for tips on being active). For blood pressure measurement and physical activity reminders, a slim majority accepted the default time (51% for each). Among those preferring tips for being active, 46% chose the default time (3 PM), with other preferred custom times between 10 AM and 12 PM. For tips for managing blood sugar, 73% chose the default option or indicated no preference. Generally, activities that occur at times that vary across individuals (medications and blood sugar checks) were most consistently seen as important for customization, whereas blood pressure measurement and physical activity reminders were less consistently seen as requiring customization. Tips elicited more varied responses; among those wanting a custom time, most preferred between 10 AM and 12 PM. Across all topics, respondents preferred to receive messages “some days” versus “every day,” except for the diabetes medication reminder—60% wanted to receive that reminder every day.

### Interviews

Rapid analysis captured overall preferences and feedback from participants. Of the 92 returned surveys, 57 (61%) indicated interest in an interview and 23 (25%) completed interviews from December 2021 to March 2022. Among the 23 interview participants, 43% were female (n=10), 40% were African American (n=9), 17% were married (n=4), 35% lived in rural areas (n=8), 26% were retired (n=6), and 52% struggled to pay basic needs (n=12).

Interviewed participants were most interested in receiving text messages on these topics: tips for healthy eating and weight management, tips from other veterans, blood sugar testing, physical activity, and preventive care reminders. They were least interested in messages related to diabetes medication and blood pressure reminders. Participants were presented with 3 hypothetical calendars showing possible timing and frequency and were asked to provide feedback on their preferred option (Figure 3). Most participants preferred Calendar A (n=12), which had the lowest frequency of messages per week. The majority indicated a preference for 1-2 text messages sent per day. Preferences for the timing of 1-way text message reminders (eg, taking medication) were found to be very specific to the individual.

The findings of these interviews directly informed the design of the protocol for the DSMS+ intervention version, allowing participants to customize their automated texting system protocol based on their interests (eg, healthy eating, weight management, physical activity, and preventative care) and to determine the timing and frequency of the messages.

### Expert Panel Review

A total of 536 messages were reviewed by 4 pairs of clinical experts. They reviewed 113 messages from existing Annie protocols, 299 messages (1-way), and 124 messages (2-way; prompts and responses from Annie based on participant responses). Overall, experts changed or rewrote 68 messages, flagged 4 messages for removal, and flagged 9 messages to be removed if the participant became pregnant while interacting with Annie. The messages flagged for removal related to type 1 diabetes or urine ketones (eg, “ANNIE-DM: Check urine or blood ketones if you have repeated blood sugars over 250 mg/dL. Call your provider if your ketones are high - they can make you sick.”). Messages flagged for pregnancy concerns were related to weight management or exercise recommendations (eg, “Annie-DM: Make it a goal to be active every day! Don't go more than 2 days without activity. https://go.usa.gov/xHwaG”). Further details on how many messages were changed, flagged, and removed are in Table 3.

**Table 3:** Results from expert panel review of text messages during the formative development of 2 diabetes self-management automated texting system interventions designed for the Annie platform within the Veterans Health Administration.

Action taken	Existing Annie protocols	1-way messages	2-way messages	Total messages
Rewritten	15	49	4	68
Flagged to be removed for all	1	3	0	4
Flagged to be removed for pregnancy concerns	1	4	4	9

### Beta Testing

Beta testing highlighted several important considerations for ensuring a successful interactive texting experience. We recognized the need for users of the final protocol to practice responding to Annie with the correct format during enrollment. Opportunities to improve message clarity were identified. In particular, some participants answered messages that included rhetorical questions, triggering an automated error message in return. Thus, messages were reworded to avoid such questions. For example, “Take off your shoes and socks! Do you notice any changes to your feet? Cuts, sores, swelling or red spots are harder to heal with diabetes.” was reworded to “Take off your shoes and socks! Notice any changes to your feet. Cuts, sores, swelling or red spots are harder to heal with diabetes.”

New outreach messages were created to address free-text responses. The messages reminded participants they could call their coordinator to change text message frequency and that their text replies were not monitored. Beta testing prompted us to add more flexibility to the timing of messages. Because some participants requested reminders at different times on different days of the week (eg, weekends vs weekdays), we made it possible for Annie to accommodate this.

Overall, beta testing results supported the acceptability of DSMS+. Participants appreciated the opportunity to change frequency, timing, or topics of texts, though they never took advantage of this opportunity during beta testing. Beta testing informed the final development of DSMS and DSMS+.

### Final Texting Protocol Description

Final components of the 2 versions of the Annie automated texting system interventions, DSMS and DSMS+, can be seen in Table 4. Receipt of the intervention requires a smartphone or cell phone and will occur over 6 months. DSMS+, by nature, will be customized to the participant’s preferences and needs through a baseline visit questionnaire. A member of the research team will then implement the customizations.

**Table 4:** Intervention elements included in the 2 diabetes self-management automated texting system interventions developed for the Annie platform within the Veterans Health Administration.

Intervention element	DSMS^a^	DSMS+^b^
Start (practice) message	Yes	Yes
Educational messages	Yes	Yes
Hyperlinks to relevant resources	Yes	Yes
Tips Written by other veterans living with diabetes (“Vet Tips”)	No	Yes
Assessment messages	No	Yes
Annie use reminders	No	Yes
Optional modules	No	Yes
Ability to customize timing and frequency	No	Yes

^a^DSMS: Diabetes Self-Management Support.

^b^DSMS+: Diabetes Self-Management Support + Interactive and Customizable Messages.

### Diabetes Self-Management Support

For DSMS, 163 educational messages were created, 85 of which linked to a video or a website. The majority of the messages provided disease education and encouraged users to be active members of their health care team. The final average Flesch-Kincaid Grade Level for all generated messages was 6.79, excluding any hyperlinks.

### Diabetes Self-Management Support + Interaction and Customizable Messages

DSMS+ contained all the messages in DSMS and 4 additional sets of messages: “Vet Tips,” assessments, Annie use reminders, and optional modules. “Vet Tips” were messages originally written by veterans living with diabetes, refined with the research team and veteran coinvestigators. If the participants sent “VETTIP YES” at any time, they received one of these tips. The tips came from surveys and interviews with veterans living with diabetes and veteran coinvestigators. Assessment messages asked participants to reflect on their management of different diabetes self-management behaviors. Annie use reminders periodically reminded users that the messages were automated or that they could call a research coordinator to make changes to their message topics and frequency. The final optional module topics were reminders for medication, blood sugar, and blood pressure, modules about foot care, physical activity, weight management, and finally, goal setting (Table 5). Many optional modules were based on existing Annie texting protocols. All messages, those in both DSMS and DSMS+, and those exclusive to DSMS+, went through the same development and expert review process.

**Table 5:** Optional modules and descriptions for the customizable diabetes self-management automated texting system intervention, Diabetes Self-Management Support + Interactive and Customizable Messages (DSMS+).

Optional module	Direction	Description
Medication reminder	1-way	Reminder to take medication at a specific times
Blood glucose reminder	2-way	Reminder to measure and send in blood glucose level
Blood glucose reminder	1-way	Reminder to measure blood glucose level
Blood pressure reminder	2-way	Reminder to measure and send in blood pressure
Foot care	1-way	Weekly tips for diabetes foot care
Physical activity	2-way	Send in minutes of physical activity for specified days
Exercise prompt	1-way	Encourages movement at specified times
Steps	2-way	Reminder to be active and later to send in step count
Weight goal setting	2-way	Asks readiness to set a weekly weight management goal
Weight management	1-way	Provides weight management tips
Diabetes self-management goal	2-way	Asks readiness to set a monthly diabetes self-management goal

## Discussion

### Principal Findings

We aimed to leverage the experience of veterans living with diabetes and clinical experts to design 2 versions of a text messaging program to support diabetes self-management. One program uses 1-way messaging to deliver diabetes self-management support without the option to customize timing or frequency (DSMS), and another delivers customizable, interactive diabetes self-management support (DSMS+). Our goal was to ensure the veteran and patient perspective was meaningfully integrated into our programs by including veterans as members of our research team, seeking feedback via surveys and interviews, and beta testing DSMS+. We also incorporated the clinician perspective by involving expert clinicians as team members and seeking feedback via a panel review.

Following this iterative, rigorous development of DSMS and DSMS+, we conducted a comparative effectiveness trial (ClinicalTrials.gov: NCT04227379). Participants were randomly assigned to either DSMS or DSMS+ to evaluate whether DSMS+ results in better outcomes for diabetes management among US veterans with uncontrolled type 2 diabetes. The primary outcome was participants’ time spent in glycemic control [30]. Secondary outcomes will include self-reported adherence to diabetes self-care recommendations, low-density lipoprotein cholesterol levels, blood pressure control, self-reported diabetes self-efficacy, and diabetes-related distress. These measures aim to capture both the clinical and psychosocial impacts of the interventions. Enrollment for this trial extended from February 2023 to April 2024, and the analysis of results is underway. By comparing the effectiveness of a customizable approach to a standard texting intervention, we seek to generate important evidence on how customization of mobile health strategies can address disparities in diabetes care within a veteran population.

### Comparison to Prior Work

A recent meta-analysis suggests that tailored or interactive ATS may be more effective than those delivering standardized content, though questions remain in regards to their development and effectiveness [15]. For example, Dobson et al [31] reported that, compared to usual care, significant improvements in HbA_1c_ were observed among those who received text messages that were tailored to their needs, goals, and demographics. In contrast, Peimani et al [32] found that, compared to those receiving standard messages, patients receiving messages customized to their disease management barriers reported greater improvements in diabetes self-care and self-efficacy, though notably not HbA_1c_. Similar preferences for customization have been documented in other Annie texting protocols outside of diabetes. For example, evaluations of 2 Annie COVID-19 protocols found that veterans desired messages that were more tailored to their demographic group, needs, and circumstances [18]. Together, this previous work has highlighted both the promise of tailoring and uncertainty about what type and extent of tailoring result in clinically meaningful health improvements.

Most prior ATS studies have focused on evaluating patient perspectives and outcomes; there has been limited description about how such interventions were developed (eg, initial drafting and integrating stakeholder feedback). This limitation may hinder others’ ability to compare across studies or reproduce similar interventions. This study aims to address this gap and adds to prior literature by describing our structured participatory process which incorporated veteran feedback and clinical expertise.

This participatory process reflects best practices in patient-centered technology-based intervention design [33,34]. Additionally, our process was guided by implementation science. For example, the Practical, Robust Implementation and Sustainability Model ( [35]) considers the characteristics of the users, intervention, and broader context to support implementation success. Tailoring message content to veteran preferences and experiences may, therefore, enhance both implementation and sustained adoption. By documenting the development process of DSMS and DSMS+, we provide a transparent account of how we developed our ATS interventions.

### Limitations

A key strength of our study is the integration of veteran perspectives and clinical expertise to ensure that the text messaging programs are clinically relevant and meaningful to veterans living with type 2 diabetes. Additionally, our study successfully engaged a diverse sample of patients in underserved settings, enhancing the relevance of the findings for those who may benefit most from diabetes self-management support. Despite these strengths, there remain limitations to consider. The low response rate for the survey may limit the generalizability of our findings, as respondents might differ significantly from nonrespondents. Additionally, our small beta testing sample may not fully capture the diverse experiences and preferences of the broader veteran population. Finally, while our veteran-informed text messaging interventions are customized to veterans, some messages (eg, Vet Tips) may not be as meaningful to nonveterans. However, the participatory design process used in our study should generalize well to other target populations.

### Conclusions

We developed 2 text messaging interventions to support diabetes self-management: a standard 1-way messaging intervention that delivers diabetes education (DSMS) and an interactive, customizable intervention designed to deliver personalized diabetes self-management support (DSMS+). By incorporating the perspectives of veterans living with diabetes and clinical experts, we aimed to create text messaging interventions that were both clinically relevant and meaningful to veterans. A subsequent trial will evaluate whether customization supports engagement and improves diabetes outcomes, offering valuable insights into the potential of tailored mobile health interventions for managing chronic diseases.

## Abbreviations

ATS: automated texting system
DSMS: Diabetes Self-Management Support
DSMS+: Diabetes Self-Management Support + Interactive and Customizable Messages
HbA1c: hemoglobin A1c
IRB: Institutional Review Board
VA: Veterans Affairs

## Data Availability

The datasets generated or analyzed during this study are not publicly available as they belong to the US Government but may be available from the corresponding author on reasonable request..
